# DDA1 promotes stage IIB–IIC colon cancer progression by activating NFκB/CSN2/GSK-3β signaling

**DOI:** 10.18632/oncotarget.7847

**Published:** 2016-03-02

**Authors:** Senlin Zhao, Huamei Tang, Dongwang Yan, Junwei Fan, Hongcheng Sun, Yugang Wen, Fudong Yu, Feifei Cui, Dongyuan Zhang, Yingming Xue, Chenchen Liu, Ben Yue, Jian Chen, Jingtao Wang, Xiao Wang, Meng Zhang, Yang Yu, Weiliang Jiang, Xisheng Liu, Yushuai Mi, Zongguang Zhou, Xuebin Qin, Zhihai Peng

**Affiliations:** ^1^ Department of General Surgery, Shanghai First People's Hospital, Affiliated to Shanghai Jiao Tong University, Shanghai, China; ^2^ Department of Pathology, Shanghai First People's Hospital, Affiliated to Shanghai Jiao Tong University, Shanghai, China; ^3^ Department of Pathology, Fudan University Affiliated Shanghai Cancer Center, Shanghai, China; ^4^ Department of Gastroenterology, Shanghai First People's Hospital, Affiliated to Shanghai Jiaotong University, Shanghai, China; ^5^ Department of Gastrointestinal Surgery, West China Hospital, Sichuan University, Chengdu, China; ^6^ Department of Neuroscience, Temple University School of Medicine, Philadelphia, PA, USA

**Keywords:** DDA1, colon cancer, tumor recurrence, prognosis, NFκB

## Abstract

Conventional high-recurrence risk factors are not sufficient to predict post-operative risk of tumor recurrence or sensitivity to 5-fluorouracil (5-FU)-based chemotherapy for stage II colon cancer. DDA1, an evolutionarily conserved gene located at 19p13.11, may be involved in the activation of nuclear factor kappaB (NFκB). This study aimed to investigate whether DDA1 contributes to tumorigenesis and progression of stage II colon cancer via activation of the NFκB pathway. We found that positive expression of DDA1 alone or in combination with p65 nuclear translocation correlated with increased risk of tumor recurrence in patients with stage IIB–IIC colon cancer. DDA1 overexpression in colon cancer lines promoted cell proliferation, facilitated cell cycle progression, inhibited 5-FU-induced apoptosis, enhanced invasion, and induced the epithelial-mesenchymal transition. Suppression of DDA1 inhibited tumor progression, and reduced tumor growth *in vivo*. We also demonstrated that DDA1-mediated tumor progression is associated with the activation of the NFκB/COP9 signalosome 2(CSN2)/glycogen synthase kinase3β (GSK3β) pathway. These results indicate that DDA1 promotes colon cancer progression through activation of NFκB/CSN2/GSK3β signaling. DDA1, together with NFκB activation status, may serve as a sensitive biomarker for tumor recurrence risk and prognosis in patients with stage IIB–IIC colon cancers.

## INTRODUCTION

Risk of tumor recurrence is clinically determined based on the pathological characteristics of the tumor. In colon cancer, the third most malignant cancer and the fourth leading cause of cancer-related mortality worldwide [1], high recurrence risk factors include tumor stage, perforation, presentation with obstruction, poor histological differentiation, lymphovascular invasion, or perineural invasion [5, 6]. Approximately 30% of colon cancer patients present with stage II cancer [2, 3]. Because some patients with stage II colon cancer benefit from post-operative adjuvant chemotherapy [4], routine 5-fluorouracil (5-FU)-based treatment is recommended only to patients with conventional high risk of tumor recurrence.

Nevertheless, approximately 20–30% of patients with stage II colon cancer undergoing 5-FU-based adjuvant chemotherapy still develop local recurrences or metachronous metastases after tumor resection [3, 7, 8]. This indicates that conventional high-risk factors cannot accurately evaluate the risk of recurrence and predict the benefit of adjuvant chemotherapy in these patients. Currently, no biomarkers except the mismatch repair (*MMR*) gene are available to differentiate patients with stage II colon cancer with a high vs. low risk of recurrence [6, 9]. This is partially due to an incomplete understanding of the molecular mechanisms of stage II colon tumorigenesis and recurrence. It is widely accepted that mutations in various genes, such as in *APC*, *KRAS*, *p53*, and *BRAF*, are involved in colon cancer tumorigenesis and progression [10–12]. These genes have also been extensively explored in stage II colon cancer for identifying the risk of tumor recurrence and for predicting chemosensitivity [13]. However, their roles in evaluating the benefits of chemotherapy following tumor relapse have not been widely explored in the clinical setting. Therefore, to better design a strategy for individualized chemotherapy, it is pivotal that researchers identify biomarkers that not only participate in colon cancer tumorigenesis and progression, but also predict chemosensitivity and the risk of tumor recurrence in patients with stage II cancer.

Emerging evidence indicates that aberrant activation of nuclear factor kappaB (NFκB) promotes tumorigenesis, progression, and chemoresistance [14]. NFκB is a transcription factor that participates in immune responses, cell proliferation, apoptosis, and cell cycle regulation [15]. Abnormal activation of NFκB is often associated with progression of many diseases, including chronic inflammation, autoimmune diseases, and cancer [16-18]. Our recently published results also indicate that canonical activation of NFκB may have a prognostic role in stage II colon cancer [19]. Gewurz, *et al.* used a genome-wide small interfering RNA (siRNA) screening approach to identify potential intrinsic mediators for activating NFκB, and found that a new gene, DET1 and DDB1 associated 1 (DDA1, also known as PCIA1), may activate NFκB via degradation of IκBα [20]. However, whether DDA1 indeed activates the NFκB pathway, thereby promoting tumorigenesis and contributing to colon cancer recurrence, has not been investigated.

DDA1 was first discovered as a gene with a 1086-bp cDNA and a 309-bp open reading frame [21]. DDA1 encodes an 11-kDa protein with 102 AA residues whose orthologs share 82–92% identity with *Arabidopsis*, invertebrates, and vertebrates [22, 23]. Binding of DDA1 to DET1 and DDB1 together results in DDD complexes, which recruit specific UBE2E enzymes such as UBE2E1, UBE2E2, and UBE2E3, to form DDD-E2 complexes [23]. A component of the DDD-E2 complexes provides a platform for interaction with Cullin4A (Cul4A) and beta-transducing (also called WD40) repeat proteins, which indicate that the complex may be involved in ubiquitination and subsequent proteasomal degradation of target proteins [22, 24]. Moreover, DDA1 was demonstrated to be a core subunit of multiple Cul4-based E3 ligases (CRLs) and may regulate CRL4s, especially in promoting cell cycle progression and DNA replication and repair [25]. In addition, DDA1 was also shown to interact with onco-proteins such as *EIF3S10*, *PSAP* and *ACTN4* [26]. These results indicate that DDA1 may be involved in tumor formation, invasion and metastasis. Whether DDA1 has prognostic value in patients with stage II colon cancer has not been assessed previously.

These results prompted us to investigate whether DDA1 participated in stage II colon cancer tumorigenesis and tumor recurrence via the modulation of NFκB/CSN2/GSK3β signaling, and could serve as a prognostic biomarker. We report that (1) DDA1 and nuclear p65 levels were increased in stage II colon cancers, (2) both DDA1 and nuclear p65 levels were significantly higher in tumors of patients with relapsed stage II cancer as compared to nonrelapsed stage II, (3) positive expression of DDA1 either alone or in combination with p65 nuclear translocation was associated with poor prognosis in stage II colon cancer, especially in stage IIB–IIC patients, and (4) DDA1 promoted proliferation, increased cell cycle S-phase arrest, inhibited 5-FU-induced apoptosis, and promoted invasion and the epithelial–mesenchyme transition (EMT) through the NFκB/CSN2/GSK3β pathway. Taken together, these results indicate that DDA1 promotes the progression of stage IIB–IIC colon cancers by activating the NFκB/CSN2/GSK-3β pathway. DDA1 may be a powerful prognostic indicator and predictor of tumor recurrence risk in patients with stage IIB–IIC colon cancer.

## RESULTS

### Overexpression of DDA1 and activation of NFκB are negatively correlated with stage IIB–IIC colon cancer patient survival

**Figure 1:**
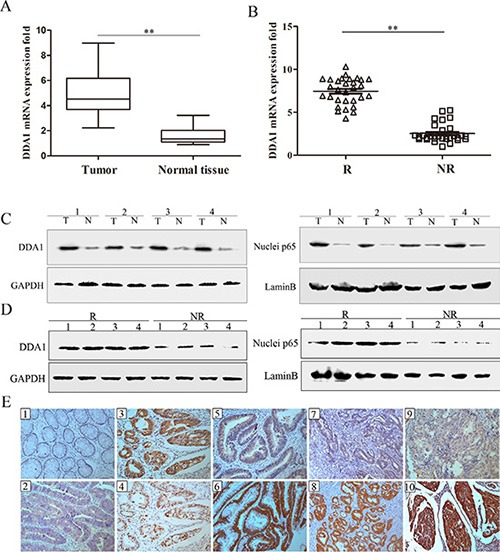
DDA1 and nuclear p65 levels in relapsed and nonrelapsed stage II colon cancer tumors (**A**) DDA1 mRNA expression was higher in tumor tissues (T) of 30 patients randomly selected from the 279 patients with stage II colon cancer than in normal tissues (N) (**P < 0.001). Glyceraldehyde 3-phosphate dehydrogenase (GAPDH) was used as a control. (**B**) DDA1 mRNA expression was higher in tumor tissues of the 30 randomly selected patients with recurring tumors (R) than nonrecurring tumors (NR) (**P < 0.001). (**C**) DDA1 and nuclear p65 protein levels were higher in the represented tumor tissues (T) than normal tissues (N). (**D**) DDA1 and nuclear p65 protein levels were higher in recurring tumors (R) than nonrecurring tumors (NR). (**E**) IHC staining of DDA1 and nuclear p65: negative DDA1 in normal tissues (E1) and positive expression in carcinoma tissues (E2). p65 was more frequently expressed in the nuclei of tumor cells that showed positive cytoplasmic DDA1 staining (E3, E4). In well-differentiated tumors of the same T3 stage, DDA1 showed weak staining in patients with non-recurrence (E5) and strong staining in patients with tumor recurrence (E6). In moderately-differentiated tumors of the same T4a stage, DDA1 showed weak staining in patients with non-recurrence (E7) and strong staining in patients with tumor recurrence (E8). In poorly-differentiated tumors of the same T4b stage, DDA1 showed weak staining in patients with non-recurrence (E9) and strong staining in patients with tumor recurrence (E10) (magnification ×200).

To investigate whether DDA1 alone or in combination with NFκB predicts the risk of tumor recurrence in patients with stage II colon cancer, levels of DDA1 and nuclear p65 were assessed. DDA1 mRNA expression was greater in the tumors of all patients with stage II colon cancer than in adjacent normal tissues of 30 randomly selected patients from the cohort (Figure 1A). DDA1 expression was significantly higher in the tumors of relapsed patients than in the tumors of nonrelapsed patients (Figure 1B). Further, DDA1 protein levels and the nuclear translocation of p65 protein, an indication of activation of NFκB, were also higher in tumor samples than in adjacent normal tissues, and in relapsed patient samples than in nonrelapsed samples (Figure 1C and 1D). Taken together, these results suggest that DDA1 upregulation and NFκB activation may be associated with both development and recurrence of stage II colon cancer.

Immunohistochemical (IHC) staining was used to quantify DDA1 levels as well as NFκB pathway activation, as measured by p65 nuclear translocation, in 101 relapsed and 178 nonrelapsed patients with stage II colon cancer. DDA1 showed mainly positive staining in the cytoplasm of tumor cells and was often accompanied by NFκB subunit p65 nuclear translocation in the tumor cells of relapsed patients with stage II colon cancer (Figure 1, 1E1–1E4). In patient tumors with the same degree of differentiation, the relapsed patients had significantly higher expression of DDA1 than nonrelapsed patients (Figure 1, 1E5–1E10). A significantly higher proportion (65.4%) of relapsed patients showed strong tumor DDA1 staining as compared to nonrelapsed patients (29.8%) (Table 1). The number of relapsed patients with weak (28.7%) and negative (5.9%) tumor DDA1 staining was significantly lower than the number of nonrelapsed patients with weak (40.4%) and negative tumor staining (29.8%) (Table 1). Relapsed patients (60.4%) exhibited higher nuclear p65 staining as compared to nonrelapsed patients (36.5%) (*P* < 0.001, Table 1). No significant association was observed between DDA1 or nuclear p65 staining and age, gender, location of the tumor, or vessel invasion (*P* > 0.05, Table 2).

**Table 1:** Correlation between DDA1or nuclei p65 staining and the tumor relapse of 279 Stage II colon cancer patients

Characteristics	No. of patients (%)	Relapse		P*
		No *n* = 178 (%)	Yes *n* = 101 (%)	
DDA1				
Negative	59 (21.1)	53 (29.8)	6 (5.9)	< 0.001
Weak	101 (36.2)	72 (40.4)	29 (28.7)	
Strong	119 (42.7)	53 (29.8)	66 (65.4)	
P65				
Negative	153 (54.8)	113 (63.5)	40 (39.6)	< 0.001
Positive	126 (45.2)	65 (36.5)	61 (60.4)	

DDA1, DET1 and DDB1 associated 1

* Chi-square test: *p* < 0.05 indicates a significant association between the variables.

**Table 2:** Relationships between clinical features and DDA1 or nuclei p65 protein expression in 279 stage II colon cancer patients

	Expression of DDA1			p*	Expression of Nucleip65		p*
	Negative *n* = 59 (%)	Weak *n* = 101 (%)	Strong *n* = 119 (%)		Negative *n* = 153 (%)	Positive *n* = 126 (%)	
Age							
< 65	27 (45.8)	46 (45.5)	54 (45.4)	0.999	73 (47.7)	54 (42.9)	0.418
≥ 65	32 (54.2)	55 (54.5)	65 (54.6)		80 (52.3)	72 (57.1)	
Gender							
Female	26 (41.1)	45 (44.6)	65 (54.6)	0.238	81 (52.9)	62 (49.2)	0.535
Male	33 (59.5)	56 (55.4)	54 (45.4)		72 (47.1)	64 (50.8)	
Location							
Right	17 (28.8)	36 (35.6)	40 (33.6)	0.29	45 (29.4)	50 (39.7)	0.111
Transverse	8 (13.6)	6 (5.9)	6 (5.0)		14 (9.2)	6 (4.8)	
Left	34 (57.6)	59 (58.5)	73 (61.4)		94 (61.4)	70 (55.5)	
pT stage							
pT3	26 (44.1)	45 (44.5)	46 (38.6)	0.001*	69 (45.1)	48 (38.1)	0.023*
pT4a	29 (49.1)	34 (33.7)	32 (26.9)		57 (37.3)	38 (30.2)	
pT4b	4 (6.8)	22 (21.8)	41 (34.5)		27 (17.6)	40 (31.7)	
AJCC stage							
IIA	26 (44.1)	45 (44.5)	46 (38.6)	0.001*	69 (45.1)	48 (38.1)	0.023*
IIB	29 (49.1)	34 (33.7)	32 (26.9)		57 (37.3)	38 (30.2)	
IIC	4 (6.8)	22 (21.8)	41 (34.5)		27 (17.6)	40 (31.7)	
Vessel invasion							
No	57 (96.6)	95 (94.1)	105 (88.2)	0.099	145 (94.8)	112 (88.9)	0.070
Yes	2 (3.4)	6 (5.9)	14 (11.8)		8 (5.2)	14 (11.1)	
Differentiation							
Well	23 (39.0)	25 (24.8)	30 (25.2)	< 0.001*	54 (35.3)	24 (19.0)	0.002*
Moderate	30 (50.8)	49 (48.5)	38 (31.9)		64 (41.8)	53 (42.1)	
Poor	6 (10.2)	27 (26.7)	51 (42.9)		35 (22.9)	49 (38.9)	
p65							
Negative	43 (72.9)	63 (62.4)	47 (39.5)	< 0.001*			
Positive	16 (27.1)	38 (37.6)	72 (60.5)				

AJCC, American Joint Committee on Cancer

* Chi-square test or Fisher's exact test: *p* < 0.05 indicates a significant relationship among the variables.

DDA1 and nuclear p65 staining were positively correlated with pT stage, American Joint Committee on Cancer (AJCC) stage and tumor differentiation in patients with stage II cancer (*P* < 0.05, Table 2). Further, 60.5% (72/119), 37.6% (38/101) and 27.1% (16/59) of patients with nuclear p65 staining exhibited high, weak, and negative DDA1 expression, respectively (*P* < 0.001, Table 2). Taken together, these results indicate that DDA1 upregulation and NFκB activation are related to recurrence in stage II colon cancer, and DDA1 contributes to the activation of NFκB.

**Figure 2:**
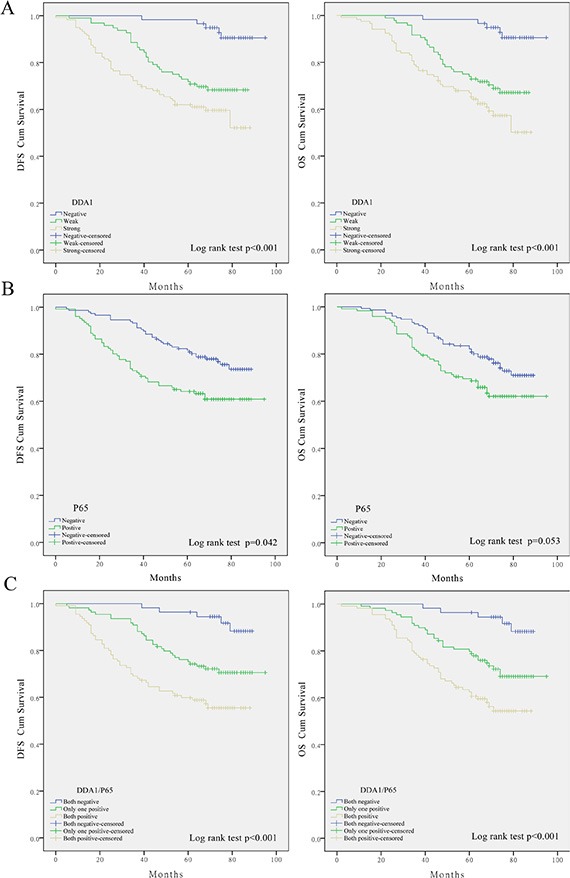
DDA1 expression predicts poor prognosis in patients with stage II colon cancer undergoing post-operative 5-FU-based adjuvant chemotherapy (**A**) Impact of DDA1 expression on patients with stage II. (**B**) Effect of nuclear p65 levels on patient DFS and OS. (**C**) Impact of DDA1 expression in combination with nuclear p65 on patient DFS and OS.

Kaplan–Meier analysis with the log-rank test was further used to investigate whether these changes are associated with patient survival. DDA1 expressing patients had lower DFS and OS than those without DDA1 expression (Figure 2A). Nuclear translocation of p65 did not significantly impact patient OS (Figure 2B). These results indicate that DDA1 has more power to predict survival than nuclear p65 translocation. However, patients with both DDA1 expression and nuclear p65 translocation had worse rates of DFS and OS than patients with either DDA1 expression or nuclear p65 translocation, and then patients negative for both (Figure 2C).

**Figure 3:**
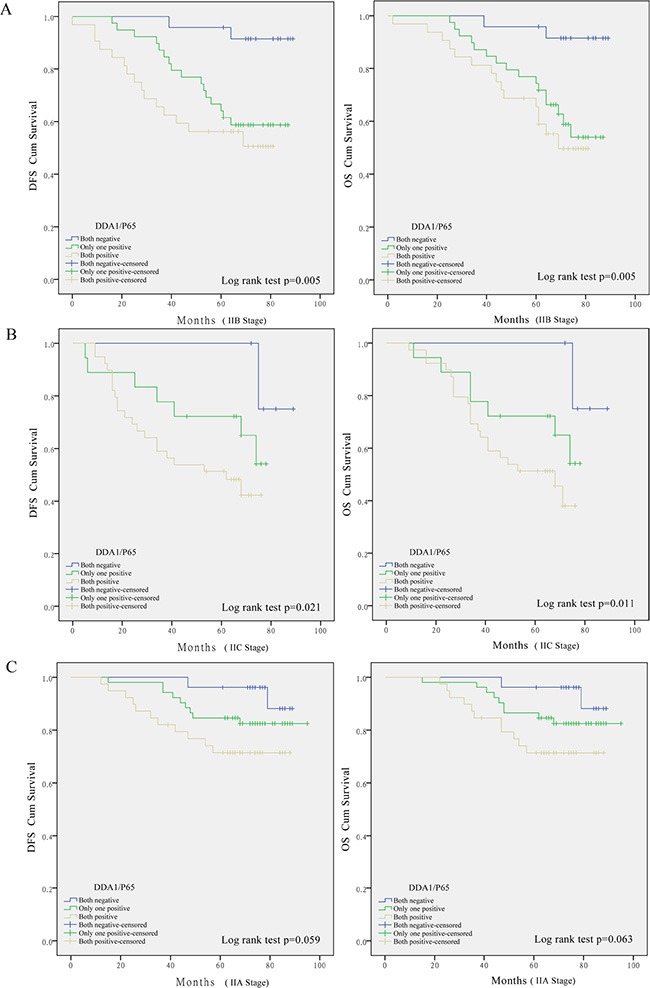
Kaplan-Meier survival analyses for stage II colon cancer patients undergoing post-operative 5-FU-based adjuvant chemotherapy Effect of DDA1 in combination with nuclear p65 expression in patients with stage IIB (**A**), stage IIC (**B**), and stage IIA (**C**) colon cancer.

These results prompted us to further investigate whether these markers have any predictive value for substages, namely, AJCC stage. DDA1 expression together with p65 nuclear translocation correlated with reduced survival in patients with stage IIB (DFS: *P* = 0.005; OS: *P* = 0.005, Figure 3A) or stage IIC colon cancer (DFS: *P* = 0.021; OS: *P* = 0.011, Figure 3B), but not in patients with stage IIA colon cancer (DFS: *P* = 0.059; OS: *P* = 0.063, Figure 3C). Univariate and multivariate analyses of DDA1 expression, nuclear p65 translocation, and clinical features such as gender and age, and clinical pathological stages further confirmed that DDA1, either alone or in combination with nuclear p65 translocation is an independent prognostic factor for high risk of tumor recurrence (Table 3).

**Table 3:** Univariate and multivariate cox proportional hazard models for overall survival (OS) and disease-free survival (DFS) in all stage II colon cancer patients

	OS				DFS			
	Univariate		Multivariate		Univariate		Multivariate	
	HR (95%CI)	p*	HR (95%CI)	p*	HR (95%CI)	p*	HR (95%CI)	p*
DDA1/p65								
Both negative	–		–		–		–	
One positive	1.73 (0.98, 8.43)	0.044*	2.17 (1.57, 4.94)	0.054	1.87 (1.23, 4.92)	0.037*	4.33 (1.59, 11.17)	0.021*
Both positive	8.77 (3.01, 15.26)	0.002*	7.06 (5.12, 14.38)	0.026*	5.69 (3.46, 10.77)	< 0.001*	6.70 (2.88, 16.05)	0.011*
DDA1								
Negative	–		–		–		–	
Weak	3.02 (1.47, 8.53)	< 0.001*	2.42 (1.35, 6.81)	0.003*	3.89 (1.87, 9.79)	0.043*	2.77 (1.09, 6.37)	0.017*
Strong	6.58 (3.42, 14.98)	0.035*	5.14 (1.46, 12.17)	0.029*	4.87 (1.74, 14.10)	< 0.001*	2.98 (1.46, 6.18)	0.007*
P65								
Negative	–		–		–		–	
Positive	1.66 (0.73, 4.99)	0.063			2.05 (1.33, 7.78)	0.002*	1.36 (0.78–7.21)	< 0.001*
Age								
< 65	–		–		–		–	
≥ 65	0.87 (0.57, 1.35)	0.541			0.87 (0.57, 1.35)	0.542		
Gender								
Female	–		–		–		–	
Male	1.28 (0.83, 1.98)	0.263			1.29 (0.84, 1.99)	0.246		
Location								
Right	–		–		–		–	
Transverse	0.93 (0.59, 1.47)	0.060			0.92 (0.58, 1.45)	0.709		
Left	0.26 (0.06, 1.06)	0.057			0.27 (0.06, 1.09)	0.065		
p T stage								
pT3	–		–		–		–	
pT4a	3.11 (1.78, 5.45)	0.021*	1.56 (0.31, 11.00)	0.046*	2.32 (1.18, 5.51)	0.029*	1.40 (0.72, 3.70)	0.032*
pT4b	6.21 (3.75, 15.29)	0.007*	3.80 (1.47, 10.36)	0.026*	4.65 (1.39, 12.07)	0.009*	2.87 (1.51, 6.48)	0.004*
AJCC stage								
IIA	–		–		–		–	
IIB	3.11 (1.78, 5.45)	0.021*	1.56 (0.31, 11.00)	0.046*	2.32 (1.18, 5.52)	0.029*	1.40 (0.72, 3.70)	0.032*
IIC	6.21 (3.75, 15.30)	0.007*	3.80 (1.47, 10.36)	0.026*	4.65 (1.39, 12.07)	0.009*	2.87 (1.51, 6.48)	0.004*
Vessel invasion								
No	–		–		–		–	
Yes	1.14 (0.62, 2.10)	< 0.001*	1.07 (0.67–3.31)	< 0.001*	2.10 (1.05, 5.18)	< 0.001*	2.26 (1.35–5.77)	< 0.001*
Differentiation								
Well	–		–		–		–	
Moderate	3.73 (1.35, 10.31)	0.013*	1.05 (0.92, 1.73)	0.031*	2.04 (1.03, 6.02)	< 0.001*	1.14 (0.97–5.52)	0.046*
Poor	8.45 (2.80, 14.52)	< 0.001*	2.16 (1.09, 5.27)	< 0.001*	4.34 (1.08, 10.22)	0.015*	2.03 (1.14–8.93)	0.034*
Chemotherapy								
5-FU/LV	–				–			
FOLFOX4	0.68 (0.36–1.05)	0.048	0.74 (0.54–1.48)	0.262	0.72 (0.52–1.20)	0.034*	0.65 (0.49–1.18)	0.067

HR: hazard ratio; CI: confidence interval.

* *p* < 0.05 indicated that the 95% CI of HR was not including 1.

### DDA1 accelerates colon cancer cell proliferation and promotes cell cycle progression

**Figure 4:**
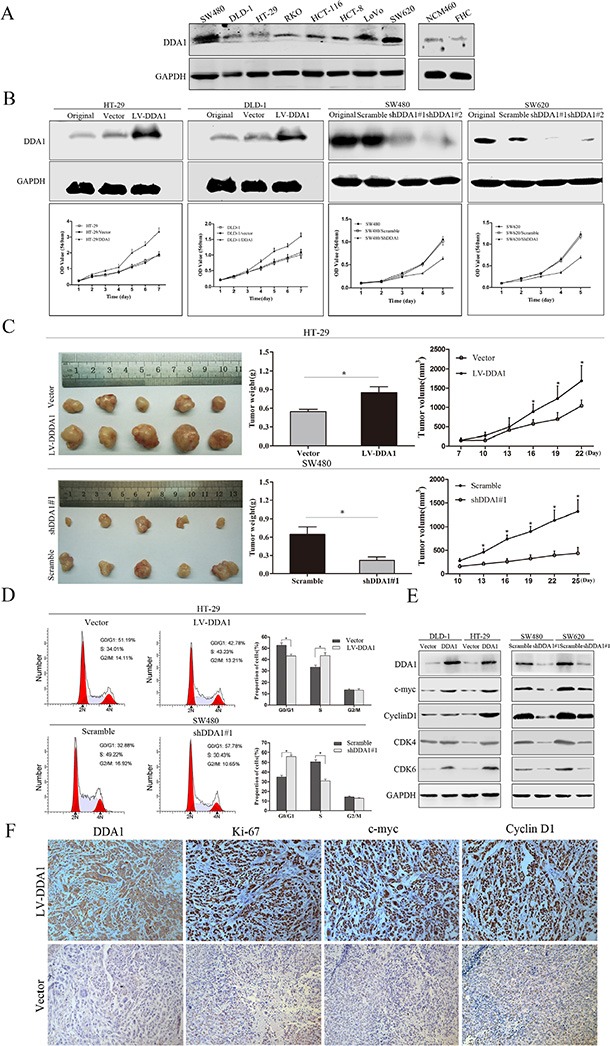
DDA1 facilitates colon cancer cell proliferation and cell cycle progression *in vitro* and *in vivo* (**A**) DDA1 expression was assessed using western blotting in eight colon cancer cell lines and two normal colon epithelial cell lines. (**B**) Overexpression or knockdown of DDA1 accelerated or inhibited the proliferation of colon cancer cells, respectively. (**C**) HT-29 and SW480 cell lines were subcutaneously injected into nude mice; tumor weight and volume growth curves are shown at three weeks post-injection (*n* = 5, *P < 0.05). (**D**) FACS analyses of cells in every phase of the cell cycle after transfection with LV-DDA1 or DDA1 shRNA#1 in HT-29 and SW480 cells (*P < 0.05). (**E**) Detection of cell cycle-related proteins in DDA1 overexpressed and knocked down cell lines. (**F**) IHC staining of DDA1, Ki-67, c-myc, and CyclinD1 in DDA1 overexpressing and control xenografts (magnification ×200).

Analysis of Gene Ontology (GO) and Kyoto Encyclopedia of Genes and Genomes (KEGG) pathways suggest that DDA1 may be involved in the development of colorectal cancer, focal adhesions, apoptosis, and activation of the NFκB signal pathway (Figure S1 and S2). Therefore, the effects of DDA1 protein on colon cancer cell proliferation and cell cycle progression were investigated using overexpression and knockdown. As no cell lines were from patients with IIB – IIC colon cancer, the protein levels of DDA1 from eight general colon cancer cell lines and two normal colon epithelial cell lines were analyzed. The DLD-1 and HT-29 cell lines with low DDA1 expression and SW480 and SW620 cell lines with high DDA1 expression were studied (Figure 4A). We over-expressed DDA1 in low-expressing lines with lentiviral expressing (LV)-DDA1 vector (LV-DDA1), or knocked down DDA1 in high-expressing lines with lentiviral expressing two different shRNAs (shDDA1#1 and shDDA1#2) (*P* < 0.05, Figure 4B). *In vivo*, the overexpression or knockdown of DDA1 in cells generated larger or smaller xenografts, respectively, as measured by tumor weights and volumes in nude mice as compared to controls (*P* < 0.05, Figure 4C). Consistently, DDA1 knockdown with shDDA1#2 suppressed the cancer cell proliferation *in vitro* and *in vivo* (Figure S3A and S3B).

As changes in cell cycle are closely related to cell proliferation, cell cycle progression was further studied in the transfected cells. Fluorescence-activated cell sorting analysis showed that DDA1 overexpression or knockdown (with shDDA1#1 or shDDA1#2) resulted in decreased G1 phase and increased S phase cells or increased G1 phase and decreased S phase cells, respectively (Figure 4D and S4A). In addition, overexpression or knockdown induced the increase or decrease, respectively, in c-myc, CyclinD1, CDK4, and CDK6 expression, indicating proliferation and G1 to S phase transition of cell cycle (Figure 4E and S4B). Further, DDA1-overexpressing xenografts from nude mice had significantly higher Ki-67, c-myc, and CyclinD1 levels than controls (Figure 4F and S5). These results indicate that DDA1 promotes tumor cell proliferation by inducing S phase arrest.

### DDA1 overexpression inhibits apoptosis in 5-FU-induced colon cancer cells

All patients with stage II colon cancer in this study received 5-FU-based adjuvant chemotherapy. We assessed whether DDA1 was upregulated by 5-FU. After treating colon cancer cell lines with different 5-FU concentrations for 48 h, DDA1 mRNA and protein levels were evaluated. There were no differences among the treated and untreated cells (data not shown), indicating that 5-FU did not upregulate DDA1 expression.

**Figure 5:**
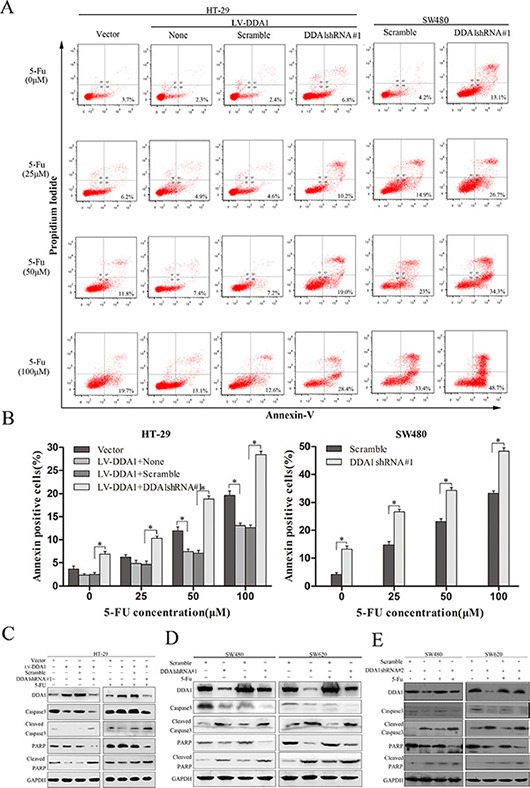
DDA1 knockdown increases apoptosis and 5-FU chemosensitivity (**A**) Apoptosis of SW620 and HT-29/LV-DDA1 cells transfected with DDA1 shRNA#1 or Scramble after treatment with 5-FU (0 μm/25 μm/50 μm/100 μm) for 48 h. (**B**) The histogram shows comparative results of Annexin-V and PI FACS for 5-FU-induced apoptosis (*P < 0.05). (**C**) Changes in caspase-3 and PARP protein levels in HT-29/LV-DDA1 cells transfected with DDA1 shRNA#1 or Scramble, with or without 5-FU (100 μm) for 48 h. (**D**) Detection of caspase-3 and PARP proteins in SW620 and SW480 cell lines transfected with DDA1 shRNA#1 or Scramble, with or without 5-FU (100 μm) for 48 h.

The effects of DDA1 on apoptosis inhibition in 5-FU-induced colon cancer cells was investigated using Annexin-V/PI staining, or by measuring apoptosis-related proteins such as cleaved caspase-3 and cleaved poly (ADP-ribose) polymerase (PARP). Annexin V/PI staining revealed that DDA1 overexpression in HT29 cells or knockdown in both DDA1 overexpressed-HT-29 cells and SW480 cells expressing DDA1 at a high level with shDDA1#1 rendered the cells resistant or sensitive to apoptosis induced by different concentrations of 5-FU, respectively (*P* < 0.05 for both, Figure 5A and 5B). In addition, the overexpression and knocked down decreased and increased 5-FU-induced caspase-3 and PARP cleavage, respectively (Figure 5C and 5D). Further, the specific target effects of the increase in the cleaved caspase-3 and PARP by shDDA1#1 were also confirmed by using the secondary shRNA, shDDA1#2 in both SW480 and SW620 cells (Figure 5E). These results show that DDA1 prohibited apoptosis in 5-FU-induced colon cancer cells and decreased chemosensitivity to 5-FU by inhibiting the activation of caspase-3 and PARP.

### DDA1 enhances invasion and induces the epithelial to mesenchymal transition

**Figure 6:**
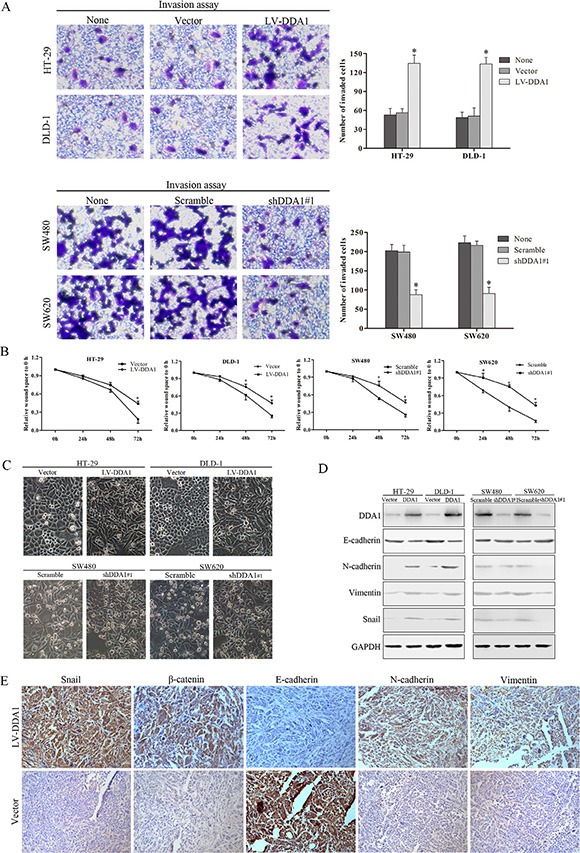
DDA1 induces colon cancer cell invasion and EMT (**A**) Overexpression or knockdown of DDA1 increased or decreased the number of invasive cells (*P < 0.05). (**B**) Wound healing assays showed wound closure was promoted in HT-29/LV-DDA1 and DLD-1/LV-DDA1 cells, and delayed in SW480/shDDA1#1 and SW620/shDDA1#1 cells compared with the vector or Scramble groups (*P < 0.05). (**C**) Exceptional expression of DDA1 led to morphological alterations in colon cancer cells (magnification ×200). (**D**) Protein expression of EMT markers in HT-29/LV-DDA1, DLD-1/LV-DDA1, SW480/DDA1 shRNA#1, SW620/DDA1 shRNA#1 cells. (**E**) IHC staining for Snail, β-catenin, E-cadherin, N-cadherin, and vimentin in nude mouse tumor xenografts. Levels of Snail, nuclear β-catenin, N-cadherin, and Vimentin increased in HT-29/LV-DDA1 tumors, whereas E-cadherin expression declined (magnification ×200).

DDA1 overexpressing cell lines (HT-29 and DLD-1) or the knocked down cells (SW480 and SW620) exhibited enhanced or reduced invasion measured using transwell invasion assay, and wound healing capacity monitored using wound healing assay, as compared to the corresponding control cells, respectively (*P* < 0.05, Figure 6A–6B and S6A–S6B). DDA1 overexpression or knockdown resulted in increased or decreased morphological transition of cells from epithelial phenotype to mesenchyme-like morphology (EMT), respectively (Figure 6C). This result indicates that DDA1 triggers the EMT in colon cancer cells.

EMT, an essential mechanism in embryonic development and tissue repair, contributes to the progression of cancer and organ fibrosis [28]. During EMT, epithelial protein E-cadherin is downregulated, while mesenchyme proteins such as N-cadherin and vimentin are upregulated [29]. Overexpression or knockdown of DDA1 resulted in decreased or increased expression of epithelial protein E-cadherin, respectively, while expression of mesenchyme proteins N-cadherin and Vimentin was increased or decreased *in vitro* (Figure 6D and S6C). Further, DDA1 overexpression upregulated Snail, an EMT inductor (Figure 6D and S7) [30]. *In vivo*, xenografted DDA1-overexpressing tumor cells had lower E-cadherin levels, but higher expression of nuclear β-catenin, N-cadherin, vimentin, and Snail than controls (Figure 6E and S7). These results suggest that DDA1 promotes invasion and migration, and induces the EMT in colon cancer cells.

### DDA1 activates NFκB/CSN2/GSK3β signaling

**Figure 7:**
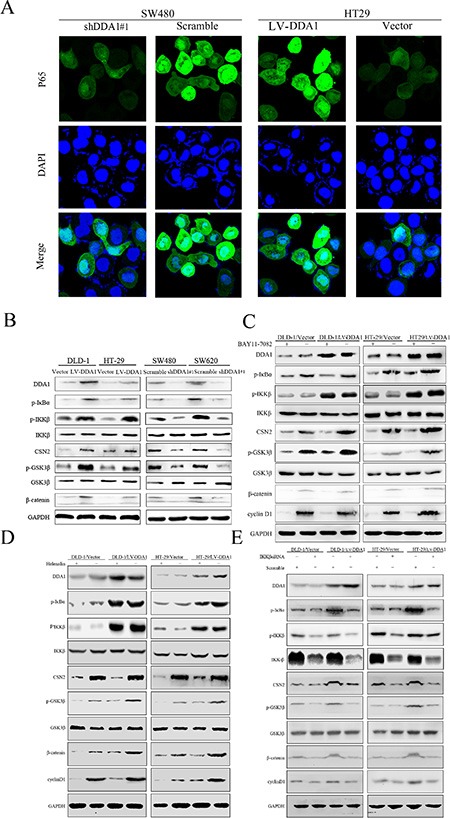
DDA1 promotes colon cancer progression by stimulating NFκB/CSN2/GSK3β signaling (**A**) Immunofluorescence analyses showed increased p65 nuclear translocation in HT-29/LV-DDA1 cells as compared with HT-29/LV-vector, but decreased in SW480/shDDA1 compared with SW480/Scramble. (**B**) Expression of DDA1/NFκB/CSN2/GSK3β pathway proteins in HT-29/LV-DDA1, DLD-1/LV-DDA1, SW480/shDDA1#1 and SW620/shDDA1#1 cells. (**C** and **D**) Changes in DDA1/NFκB/CSN2/GSK3β signaling proteins in HT-29/LV-DDA1 and DLD-1/LV-DDA1 cells after treatment with BAY-11-7082 and Helenalin. (**E**) Changes in DDA1/NFκB/CSN2/GSK3β pathway proteins in HT-29/LV-DDA1 and DLD-1/LV-DDA1 cells after transfection with IKKβ siRNA and Scramble.

Classical activation of NFκB is involved in tumor proliferation, anti-apoptosis, and EMT [15, 28]. In addition, NFκB activation is required for the induction of the CSN2/GSK3β pathway, which enhances tumor invasion and metastasis [31]. CSN2, the second and most conserved subunit of the COP9 signalosome in eukaryotes, is also targeted by NFκB [32]. Therefore, we investigated whether DDA1 activates NFκB/CSN2/GSK3β signaling. We found that DDA1 overexpression or the knockdown with shDDA1#1 increased or decreased nuclear p65 translocation, indicating classical activation of NFκB, respectively (Figure 7A). We also demonstrated that the DDA1 overexpression or knockdown with shDDA1#1 promoted or inhibited IKKβ phosphorylation, thereby upregulating p-IκBα, which resulted in nuclear p65 translocation via degradation of p-IκBα, respectively (Figure 7B). These results indicate that DDA1 activates the classical NFκB pathway by increasing p-IKKβ expression.

**Figure 8:**
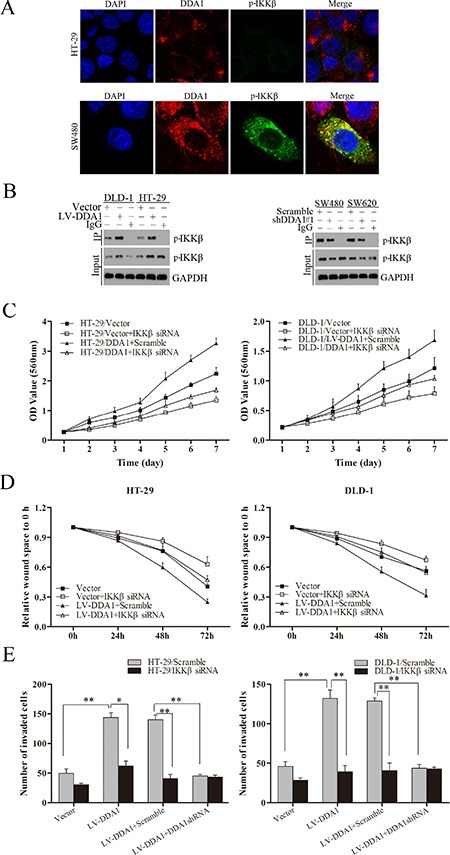
DDA1 activates the NFκB/CSN2/GSK3β pathway by regulating IKKβ phosphorylation (**A**) Co-expression assays in HT-29 and SW480 cells: DDA1 (red), p-IKKβ (green), DAPI nuclear staining (blue). Merged images of DDA1 (red) and p-IKKβ (green) with DAPI (blue) are also shown. (**B**) Co-immunoprecipitation between endogenous DDA1 and p-IKKβ in DLD-1, HT-29, SW480, and SW620 cells. p-IKKβ was detected in the immunoprecipitate using an anti-DDA1 antibody as a bait. (**C**) *In vitro* proliferation assays in LV-DDA1-transfected or vector-transfected cells after transfection with IKKβ siRNA. (**D**) Wound healing assays were performed in LV-DDA1-transfected or vector-transfected cells after transfection with IKKβ siRNA. (**E**) Transwell invasion assays with LV-DDA1-transfected or vector-transfected cells after transfection with IKKβ siRNA.

p65 nuclear translocation reportedly induces CSN2 upregulation followed by phosphorylation of GSK3β at serine 9, which promotes tumor metastasis and EMT [31]. Overexpression or knockdown of DDA1 resulted in up- or downregulation of CSN2 and p-GSK3β, respectively (Figure 7B). Increased CNS2 and p-GSK3β levels induced by DDA1 overexpression were markedly attenuated following treatment with BAY-11-7082, an inhibitor of phosphorylation and degradation of IκBα, Helenalin, a p65 inhibitor, or siRNA specific for IKKβ (Figure 7C–7E). To directly assess whether DDA1 activated NFκB/CSN2/GSK3β signaling by regulating IKKβ expression, DDA1 and p-IKKβ were analyzed by immunofluorescence. DDA1 was co-expressed with p-IKKβ (Figure 8A), directly bound to p-IKKβ and stabilized its expression (Figure 8B). Functionally, silencing IKKβ with IKKβ siRNA inhibited the increased proliferation, migration, and invasion promoted by DDA1 (Figure 8C–8E). Lastly, the specific target effects of the DDA1-mediated p65 nuclear translocation and p-IKKβ stabilization were further confirmed by using the secondary shRNA shDDA1#2 to inhibit DDA1 activity in both SW480 and SW620 cells (Figure S8A and S8B). These results indicate that DDA1 promotes colon cancer cell proliferation, migration, and invasion by activating NFκB/CSN2/GSK3β signaling. DDA1 specifically promotes and stabilizes p-IKKβ, which increases p-IκBα, p65 nuclear translocation, and expression of CNS2 and p-GSK3β.

## DISCUSSION

We observed that DDA1 predicts tumor recurrence and poor prognosis in patients with stage II colon cancer, especially for patients with stage IIB–IIC disease undergoing post-operative chemotherapy with 5-FU/LV or FOLFOX4. DDA1 overexpression inhibited apoptosis in 5-FU-induced colon cancer cells, although DDA1 was not upregulated by 5-FU treatment. These findings highlight the critical role of DDA1 in evaluating the risk of tumor recurrence and chemosensitivity to 5-FU in patients with stage II colon cancer. Further, these results suggest that patients with stage IIB–IIC colon cancer with DDA1-positive expression have a high risk of recurrence and should be considered for stronger chemotherapy regimens. Although the retrospective studies reported here were performed with only 279 patients, they provide a strong justification for large-scale retrospective and prospective studies in the future.

Tumor cell proliferation and cell cycle changes play critical roles in post-operative tumor relapse [33]. In the present study, we found that DDA1 promoted colon cancer cell proliferation and positively modulated cell cycle proteins including Ki-67, CyclinD1 and CDK4/6 *in vitro* and *in vivo*, which resulted in cell cycle S-phase arrest. These results indicate that DDA1 plays a critical role in tumorigenesis in stage II colon cancer. These findings are in agreement with the current view that oncogenes enhance tumorigenesis by influencing cell cycle progression [34]. For example, knockdown of the tumor genes such Cul4A, Cul4B, and Wdr23 increased double-stranded DNA breaks following changes in cell cycle [35, 36]. Double-stranded DNA breaks in DDA1^−/−^ cells increased in a similar manner as in Cul4A^−/−^, Cul4B^−/−^, and Wdr23^−/−^ cells [25], which play important roles in the proliferation of cells and cell cycle modulation.

It is widely accepted that tumor invasion and metastasis are the main driving forces behind tumor recurrence and chemoresistance [37]. In the present study, DDA1 overexpression was positively associated with T stage, AJCC stage, and differentiation in patients with stage II colon cancer. More importantly, DDA1-positive expression incidence was significantly higher in the recurrent tumor group than in the non-recurrent group. DDA1 overexpression enhanced the invasive and wound-healing capabilities of colon cancer cell lines. In addition, it is widely accepted that EMT is an important process during embryonic development, invasion, metastasis, and chemoresistance [38]. We demonstrated here that DDA1 overexpression promotes the EMT of colon cancer cells with changes in cell morphology, along with downregulation of the epithelial protein E-cadherin and upregulation of the mesenchyme molecules N-cadherin and Vimentin. These findings provide further evidence to support the hypothesis that DDA1 promotes tumor recurrence in patients with stage II colon cancer by enhancing the invasive and metastatic abilities of cancer cells.

NFκB transcription factors are homodimers or heterodimers of NFκB1 (p50), NFκB2 (p52), RelA (p65), RelB, or cREL [39], and have been widely reported to participate in tumor initiation, cell proliferation, apoptosis, chemoresistance, and the EMT [28, 40-42]. In the canonical NFκB pathway, IKKβ is activated with increased expression of p-IKKβ. NFKBIA (IκBα), NFKBIB (IκBβ) and NFKBIE (IκBε) are then phosphorylated, leading to their proteasomal degradation and p65 nuclear translocation [44]. In the present study, DDA1-positive expression was generally accompanied by p65 nuclear translocation. With increased DDA1 expression, rates of p65 nuclear translocation increased gradually. Furthermore, DDA1 alone or in combination with nuclear p65 positive expression predicted poorer DFS and OS, especially in patients with stage IIB-IIC colon cancer. These discoveries indicate that the underlying mechanism by which DDA1 promotes stage II colon cancer progression is related to canonical activation of the NFκB pathway. p65 nuclear translocation stabilized the expression of Snail and inhibited its phosphorylation and ubiquitination by blocking its binding to GSK3β, and inducing CSN2 and the EMT [31]. Mechanistically, we found that DDA1 promoted proliferation and invasion in colon cancer cells by enhancing and stabilizing p-IKKβ and triggering the phosphorylation and degradation of IκBα followed by the activation of NFκB/CSN2/GSK3β signaling. Consistently, these effects are suppressed by treatment with inhibitors of the phosphorylation and degradation of IκBα or of p65 nuclear translocation, or transfection with siRNAs specific for IKKβ. Importantly, the molecular mechanisms by which DDA1 increases and stabilizes the expression of p-IKKβ remain unclear. Further investigation is required to determine whether DDA1 activates the canonical NFκB pathway through other approaches.

Notably, only 50% of the patients in this study with stage II colon cancer exhibited DDA1-positive expression in combination with p65 nuclear translocation (Table 2). These data highlight that in addition to the NFκB pathway, other signaling pathways may also be involved in DDA1-related tumorigenesis [45]. This interpretation is further supported by the analysis of GO and KEGG pathways and the observation that DDA1 also activates other signal pathways in colon cancers (Figure S1 and S2).

In summary, DDA1 promotes the progression of stage II colon cancer through the activation of the NFκB/CSN2/GSK3β pathway. DDA1, together with NFκB status, can aid in determining the risk of tumor recurrence in patients with stage II colon cancer, and may be a potential marker to evaluate chemosensitivity to 5-FU. These findings could contribute to improved personalized chemotherapy options for patients with stage IIB–IIC colon cancer. However, the cell lines used in the present study were not specifically from patients with stage II colon cancer. More investigations using stage II colon cancer cell lines and large-sample clinical trials are needed. In addition, the potential for use of DDA1 as a prognostic indicator in patients with other stages of colon cancer should also be explored.

## MATERIALS AND METHODS

### Patients and tissue samples

Tissue specimens were obtained from 279 patients with stage II colon cancer (Table S1) who underwent tumor resection at Shanghai Jiaotong University Affiliated First People's Hospital, Shanghai, China, between 2001 and 2007. All patients were categorized into tumor recurrence and non-recurrence groups based on the recurrent tumor conditions. None of the patients underwent anticancer treatment before surgery. Diagnoses of all patients were confirmed without microsatellite instability high (MSI-H) pathologically. All patients had at least one conventional high-risk factor for tumor recurrence and received 5-FU-based chemotherapy (5-FU/LV or FOLFOX4; Table S2) after surgery according to National Comprehensive Cancer Network guidelines. All patients signed informed consent forms before enrolling in the study. The study was approved by the Institutional Research Ethics Committee of the hospital.

### Cell culture, reagents, and transfection

Cells (SW480, DLD-1, HT-29, RKO, HCT116, HCT8, LoVo, SW620, NCM460, FHC) used in this study were purchased from the American Type Culture Collection. The cells were cultured in Dulbecco's modified Eagle's medium (DMEM) (GIBCO, Grand Island, NY) supplemented with 10% fetal bovine serum (GIBCO), 1% penicillin, and streptomycin with humidity at 37°C and 5% CO_2_. Cell lines overexpressing DDA1 were constructed by transfection with lentiviral vectors encoding human DDA1 (LV-DDA1), and DDA1 knockdown cell lines were established through transfection with either shDDA1#1 or shDDA1#2. IκB kinase complex β (IKKβ) was knocked down by transfection with siRNA IKKβ plasmids. LV-vector and LV-Scramble were used as normal controls. All interference sequences are shown in Table S3.

### qRT-PCR, western blotting, and immunohistochemical staining

Quantitative reverse transcription-polymerase chain reaction (qRT-PCR), western blotting, and immunohistochemical (IHC) staining were performed, and IHC staining scores were evaluated as described previously [19]. Primers used in qRT-PCR are summarized in Table S4. Antibodies are provided in Table S5.

### *In vitro* proliferation, invasion and wound healing assays

For proliferation assays, transfected HT-29, DLD-1, SW480 and SW620 cells were seeded at 1 × 10^5^ cells per well into 96-well plates. Cell Counting Kit-8 (Beyotime, Biotechnology, Jiangsu, China) was used to test absorbance at 560 nm for each well at different time points using a micro-plate reader (Bio-Rad, Hercules, CA).

To determine the effects of DDA1 on invasion, 1 × 10^5^ cells were seeded onto matrigel-coated chambers (BD Biosciences, San Jose, CA), with serum-free media in the upper chambers and complete media in the lower chambers, and incubated for 24 h. After fixing and staining with 0.1% crystal violet, stained cells were counted and photos were taken using a light microscope (Olympus Corporation, Center Valley, PA).

In wound healing assays, cells were plated into six-well plates. Wounds were scraped when cells reached 90% confluence. The cells were washed with phosphate-buffered saline (PBS) and wound widths were measured and analyzed at 0, 24, 48 and 72 h.

### Immunofluorescence analysis

Cells were plated on glass-bottom dishes and incubated overnight. After washing with PBS three times, cells were fixed by 4% paraformaldehyde for 15 min and washed three times with PBS. Cells were permeabilized with 0.1% Triton X-100 for 10 min, followed by incubation with p65 primary antibody overnight at 4°C. Cells were then incubated with Alexa Fluor 488-conjugated secondary antibody (Santa Cruz Biotechnology, Santa Cruz, CA) for 2 h at room temperature. 4′,6-Diamidino-2-phenylindole (DAPI) (Roche, Basle, Switzerland) was used to stain nuclei. Fluorescence images were collected using a confocal laser-scanning microscope (TCS SP8; Leica, Wetzlar, Germany).

### Flow cytometry assay for cell cycle and apoptosis

Cells were collected at the logarithmic stage of growth, and were centrifuged and then resuspended at 2 × 10^6^ cells/mL. After 30 min incubation in 80% ethanol at 4°C, propidium iodide (PI) staining was utilized to measure cell cycle stage using flow cytometry (BD Biosciences). The Annexin V-APC/PI Apoptosis Kit (eBioscience, San Diego, CA) was used for the apoptosis assay according to the manufacturer's instructions. Flow cytometery was performed to determine cell number, and data were analyzed using FlowJo9.1 software (Tree Star Inc., Ashland, OR).

### Co-immunoprecipitation assay

HT-29 and DLD-1 cell lines were transfected with empty vector or LV-DDA1 plasmids. SW480 and SW620 cell lines were transfected with Scramble or DDA1 shRNA plasmids. Cell lysate was immunoprecipitated with a DDA1 antibody. The p-IKKβ antibody was used to detect the level of p-IKKβ.

### Xenografted tumors of nude mice

Colon cancer cells HT-29/vector, HT-29/LV-DDA, SW480/Scramble, and SW480/shDDA1#1 (1 × 10^7^ cells in 100 μL of DMEM) were injected subcutaneously into the flanks of nude mice (4 week old, male, Shanghai SLAC Laboratory Animal Co., Ltd., Shanghai, China). Tumor weights and volumes were measured every three days. All mice were killed after four weeks, and tumor samples were harvested and embedded in paraffin. Procedures involving animals conformed to the guidelines of the Institutional Animal Care and Use Committee of Shanghai Jiaotong University Affiliated Shanghai First People's Hospital.

### Statistical analysis

Chi-square test or Fisher's exact test was used to compare the differences in categorical variables. Student's *t*-test or one-way analysis of variance was used to analyze the differences in continuous variables. Kaplan-Meier analyses with log-rank tests were used to evaluate disease-free survival (DFS) and overall survival (OS). The Cox proportional hazard model was performed to estimate the hazard ratio and 95% confidence intervals for DFS and OS. SPSS 19.0 statistical software (SPSS Inc., Chicago, IL) was used to analyze all data. *P* < 0.05 was regarded as statistically significant.

## SUPPLEMENTARY MATERIALS FIGURES AND TABLES


