# Supplementary material for: DDA1 promotes stage IIB–IIC colon cancer progression by activating NFκB/CSN2/GSK-3β signaling
Source: Oncotarget. 2016 Mar 2;7(15):19794–812. doi: 10.18632/oncotarget.7847 (PMC4991419; doi:10.18632/oncotarget.7847)
Supplement: Supplementary file 1 [file oncotarget-07-19794-s001.pdf]

DDA1 promotes stage IIB–IIC colon cancer progression by activating NFκB/CSN2/GSK-3β signaling

Supplementary Materials

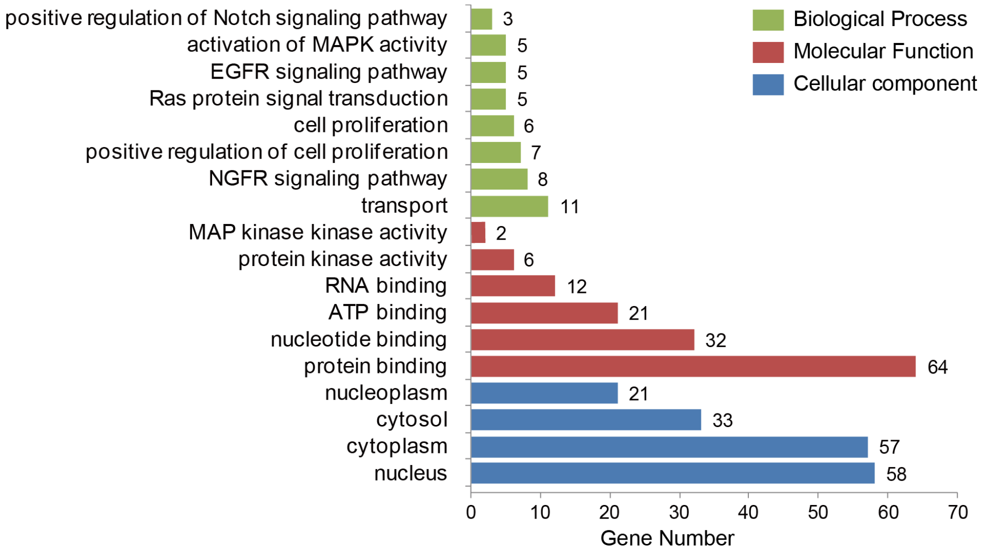

Supplementary Figure S1: DDA1.coexpression.gene.GO annotation GeneCodis.

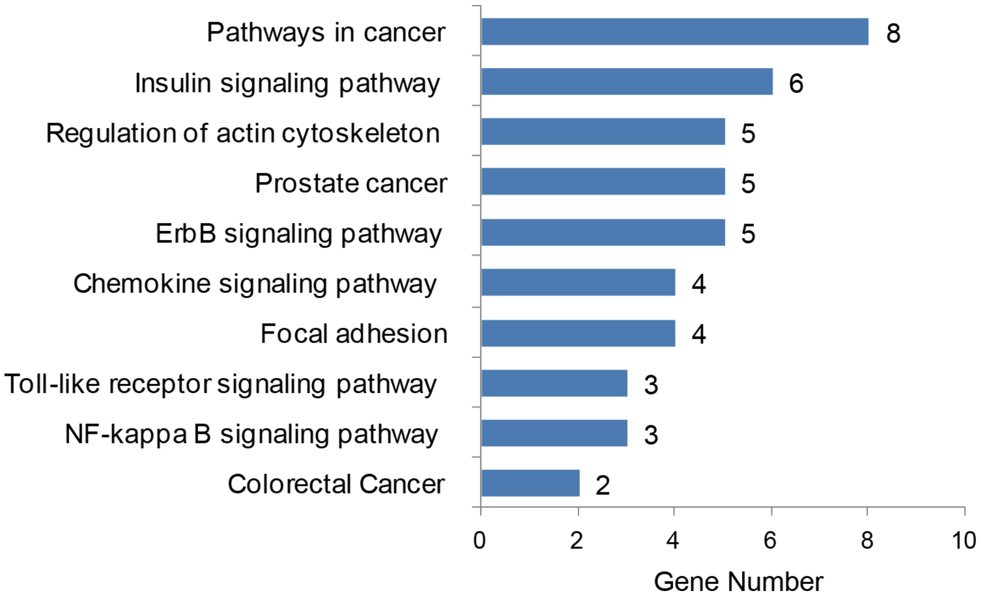

Supplementary Figure S2: DDA1.coexpression.gene.KEGG.GeneCodis.

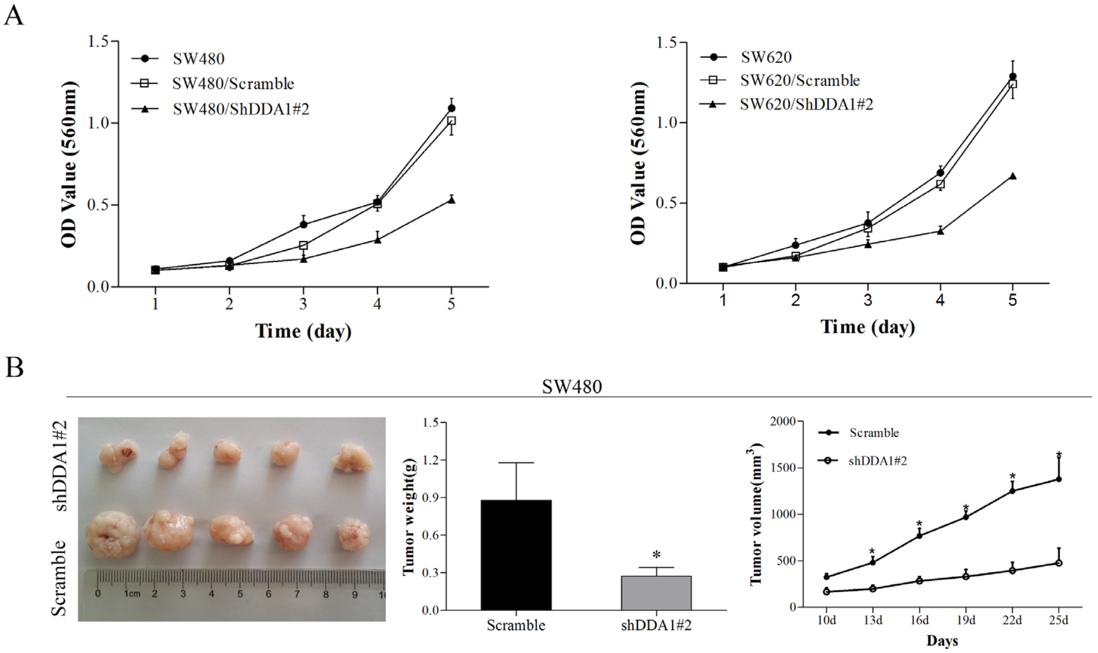

Supplementary Figure S3: DDA1 knockdown by transfecting with shDDA1#2 inhibited the proliferation of colon cancer cells *in vitro* and *in vivo*. (**A**) DDA1 knockdown with shDDA1#2 inhibited SW480 and SW620 cell proliferation *in vitro*, as compared with controls. (**B**) DDA1 knockdown in SW480 cells prohibited xenografted tumor growth in nude mice, compared with the scramble group. Tumor weight and volume growth curves are shown at three weeks post-injection ($n = 5$, $^*P < 0.05$)

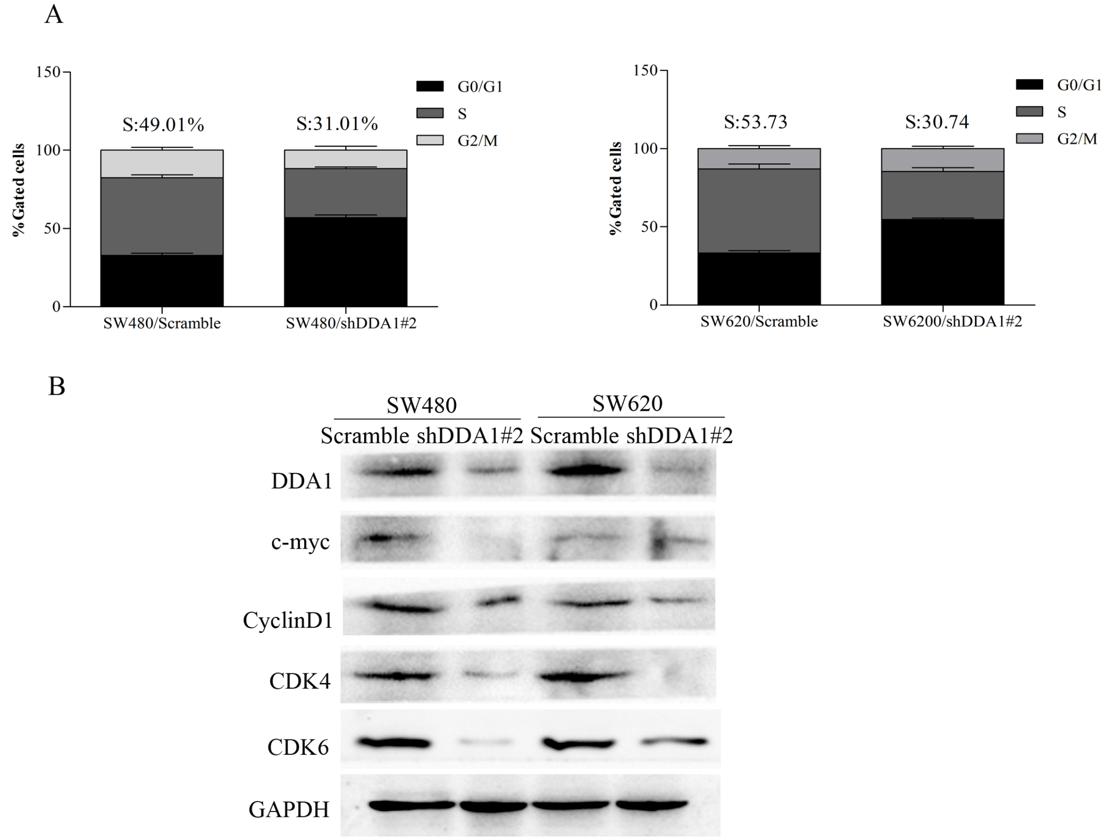

Supplementary Figure S4: DDA1 knockdown prohibited the cell cycle G0/G1 to S–phase transition. (**A**) FACS analyses of cells in every phase of cell cycle after transfection with shDDA1#2 in SW480 and SW620 cell lines; knockdown inhibited cell cycle S-phase arrest ($^*P < 0.05$). (**B**) Western blotting shows cell cycle-related proteins in DDA1 knockdown cell lines.

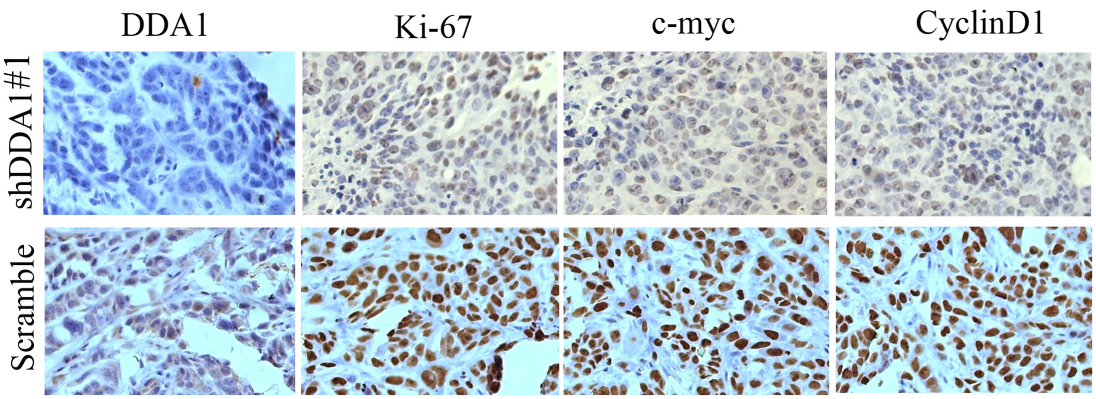

Supplementary Figure S5: IHC staining of DDA1 and cell cycle-associated markers in DDA1 knockdown xenografts and controls. DDA1 knockdown in SW480 cells inhibited Ki-67, c-myc, and CyclinD1 expression in xenografts as compared with the control group (n = 5, magnification ×200).

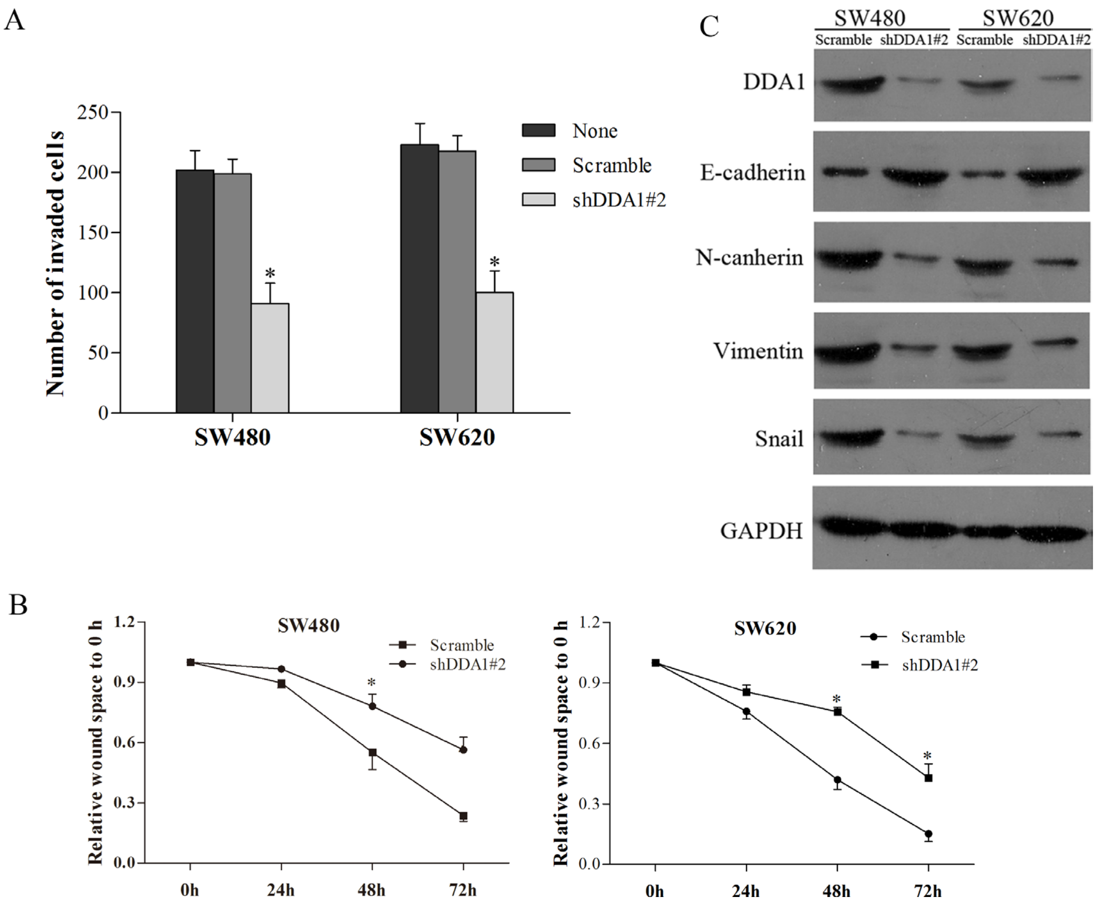

Supplementary Figure S6: DDA1 knockdown prohibited invasion and reversed the EMT in colon cancer cells. (**A**) Invasion assays showed that DDA1 knockdown in SW480 and SW620 cells inhibited cell invasion (*$P < 0.05$). (**B**) Wound healing assays showed wound closure was delayed in SW480/shDDA1#2 and SW620/shDDA1#2 cells compared with the scramble groups (*$P < 0.05$). (**C**) EMT marker protein expression in SW480/DDA1 shRNA#2, and SW620/DDA1 shRNA#2 cells.

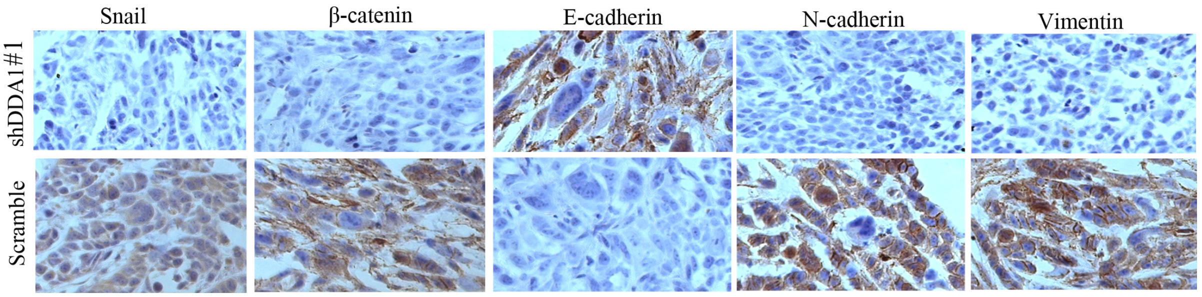

Supplementary Figure S7: IHC staining of DDA1 and EMT-associated markers in xenografts. DDA1 knockdown decreased Snail, β-catenin, N-cadherin, and vimentin expression in subcutaneous xenografted tumors of nude mice, and increased E-cadherin expression (n = 5, magnification ×200).

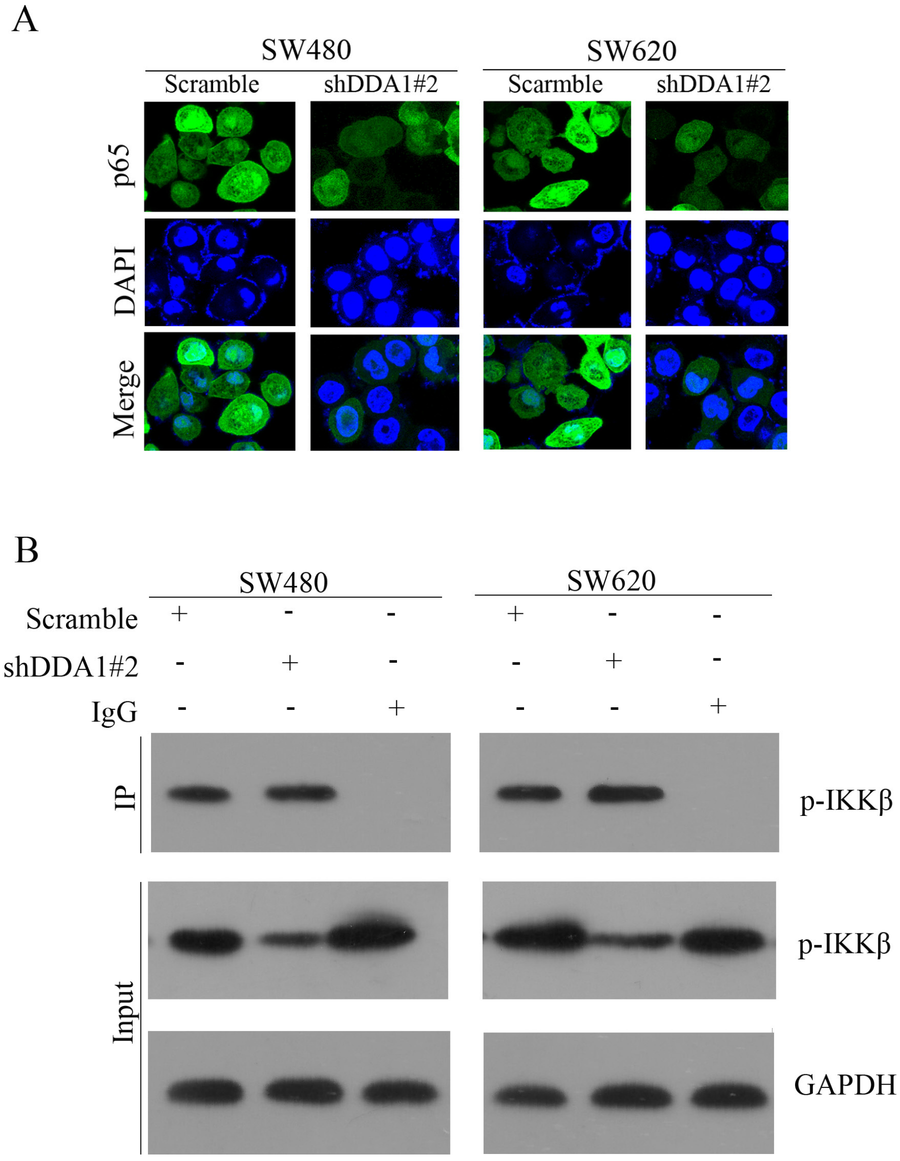

Supplementary Figure S8: DDA1 knockdown inhibited NFκB activation. (**A**) Immunofluorescence analyses showed p65 nuclear translocation decreased in SW480/shDDA1#2 and SW620/shDDA1#2 cells as compared with the Scramble group. (**B**) Co-immunoprecipitation of endogenous DDA1 and p-IKKβ in SW480/shDDA1#2, SW620/shDDA1#2 cells and their compared group. After using anti-DDA1 antibody as bait, p-IKKβ was detected in the immunoprecipitate.

Supplementary Table S1: Clinicopathologic characteristics of 279 stage II colon cancer patients

Characteristics	No. of patients (%)	Relapse	
		No<br>*n* = 178 (%)	Yes<br>*n* = 101 (%)
Age			
< 65	127 (45.5)	80 (44.9)	47 (46.5)
≥ 65	152 (54.5)	98 (55.1)	54 (53.5)
Gender			
Female	136 (48.7)	82 (46.1)	54 (53.5)
Male	143 (51.3)	96 (53.9)	47 (46.5)
Location			
Right	95 (34.0)	56 (31.5)	39 (38.6)
Transverse	20 (7.2)	15 (8.4)	5 (5.0)
Left	164 (58.8)	107 (60.1)	57 (56.4)
p T stage			
pT3	117 (41.9)	95 (54.4)	22 (21.8)
pT4a	95 (34.1)	55 (30.9)	40 (39.6)
pT4b	67 (24.0)	28 (15.7)	39 (38.6)
AJCC stage			
IIA	117 (41.9)	95 (54.4)	22 (21.8)
IIB	95 (34.1)	55 (30.9)	40 (39.6)
IIC	67 (24.0)	28 (15.7)	39 (38.6)
Vessel invasion			
No	257 (92.1)	170 (95.5)	87 (86.1)
Yes	22 (7.9)	8 (4.5)	14 (13.9)
Differentiation			
Well	78 (28.0)	58 (32.6)	20 (19.8)
Moderate	117 (41.9)	77 (43.3)	40 (39.6)
Poor	84 (30.1)	43 (24.1)	41 (40.6)
Chemotherapy			
5-FU/LV	113 (40.5)	74 (41.6)	39 (38.6)
FOLFOX4	166 (59.5)	104 (58.4)	62 (61.4)

AJCC, American Joint Committee on Cancer; 5-FU, 5-fluorouracil; LV, leucovorin; FOLFOX4, 5-fluorouracil/5-fluorouracil /leucovorin/oxaliplatin.

Supplementary Table S2: Postoperative 5-FU-based adjuvant chemotherapy plans for patients with stage II colon cancer

Regimens	Dose	Administration	Time for drug	Interval cycles	Cycles
**Mayo Clinic regimen**					
LV	500 mg/m^2	i.v. (2 h Inf)	Day 1	Every 8weeks	Four-week cycles
5-Fu	500 mg/m^2	i.v.after LV1 h	Day 1		
**FOLFOX4 regimen**					
5-Fu	400 mg/m^2	i.v. (bolus)	Day 1 + 2	Every 2 weeks	Six-week cycles
5-Fu	600 mg/m^2	i.v. (22 h Inf)	Day 1 + 2		
LV	200 mg/m^2	i.v. (2 h Inf)	Day 1 + 2		
Oxaliplatin	85 mg/m^2	i.v. (2 h Inf)	Day 1		

5-Fu: 5-fluorouracil; i.v.: intravenous; LV, Leucovorin.

Supplementary Table S3: Interference sequences used in this study

Name	Sequence
shDDA1#1	CGGACTGCCTGTCTACAACAAA
shDDA1#2	AGCCCTCAGTCTACCTGCCTACT
IKKβsiRNA	TGATGAATCTCCTCCGAAA

Supplementary Table S4: Primer sequences used for qPCR in this study

Gene name (Human)	Primer sequence (5′-3′)	
	Forward	Reverse
DDA1	TTTAGTCGATTTCACGCGGAC	ATCTGTTCAGACGGGTACTCG
P65	ATGGGCAAGTCAGCTTCCAAA	GCCTCAGAATACTGTTGAGCCT
GAPDH	TGTGGGCATCAATGGATTTGG	ACACCATGTATTCCGGGTCAAT
LaminB	AGGAACCCGAGTTCAGCTAC	CACGTCGAGGTCACCGAAAG

Supplementary Table S5: Antibodies applied in this study

Antibody	WB	IHC	IF	Specificity	Company
DDA1 (ab189975)	1:1000	1:400	–	Rabbit polyclonal	Abcam
CyclinD1 (sc-246)	1:500	1:100	–	Mouse monoclonal	Santa Cruz Biotechnology
C-myc (BS2462)	1:500	1:200	–	Rabbit polyclonal	Biowold
CDK4 (sc-260)	1:500	–	–	Rabbit polyclonal	Santa Cruz Biotechnology
CDK6 (sc-7961)	1:500	–	–	Mouse monoclonal	Santa Cruz Biotechnology
Ki-67 (#12202)	–	1:100	–	Mouse monoclonal	Cell Signaling Technology
P65 (#8242)	–	1:800	1:400	Rabbit monoclonal	Cell Signaling Technology
β-catenin (#8480)	1:1000	1:200	–	Rabbit monoclonal	Cell Signaling Technology
E-cadherin (sc-21791)	1:500	1:100	–	Mouse monoclonal	Santa Cruz Biotechnology
N-cadherin (ab18203)	1:1000	1:100	–	Rabbit polyclonal	Abcam
Vimentin (sc-53464)	1:500	1:100	–	Mouse monoclonal	Santa Cruz Biotechnology
Snail1 (BS1853)	1:500	1:200	–	Rabbit polyclonal	Bioworld
p-IκBα (#9246)	1:1000	–	–	Mouse Monoclonal	Cell Signaling Technology
IκBα (#4812)	1:1000	–	–	Rabbit Monoclonal	Cell Signaling Technology
p-GSK3β (BS4084)	1:500	–	–	Rabbit polyclonal	Bioworld
GSK3β (BS1402)	1:500	–	–	Rabbit monoclonal	Bioworld
GAPDH (ab181602)	1:10000	–	–	Rabbit monoclonal	Abcam
Caspase 3 (#9665)	1:1000	–	–	Rabbit monlclonal	Cell Signaling Technology
Cleaved Caspase3 (#9664)	1:1000	–	–	Rabbit monlclonal	Cell Signaling Technology
PARP (ab6079)	1:400	–	–	Rabbit polyclonal	Abcam
